# The Environmental Impact of the Athlete’s Plate Nutrition Education Tool

**DOI:** 10.3390/nu12082484

**Published:** 2020-08-18

**Authors:** Alba Reguant-Closa, Andreas Roesch, Jens Lansche, Thomas Nemecek, Timothy G Lohman, Nanna L Meyer

**Affiliations:** 1 International Doctoral School, University of Andorra, Andorra, AD600 Sant Julià de Lòria, Andorra; 2 Agroscope, Life Cycle Assessment Research Group, CH-8046 Zurich, Switzerland; andreas.roesch@agroscope.admin.ch (A.R.); jens.lansche@agroscope.admin.ch (J.L.); thomas.nemecek@agroscope.admin.ch (T.N.); 3 Emeritus Faculty, University of Arizona, Tucson, AZ 85711, USA; timglohman@gmail.com; 4 Beth-El College of Nursing and Health Sciences, Department of Human Physiology and Nutrition, William J. Hybl Sports Medicine and Performance Center, University of Colorado, Colorado Springs, CO 80918, USA; nmeyer2@uccs.edu

**Keywords:** sports nutrition, protein, periodized nutrition, environmental impact, nutrition education, sustainability, life cycle assessment

## Abstract

Periodized nutrition is necessary to optimize training and enhance performance through the season. The Athlete’s Plate (AP) is a nutrition education tool developed to teach athletes how to design their plates depending on training load (e.g., volume × intensity), from easy (E), moderate (M) to hard (H). The AP was validated, confirming its recommendations according to international sports nutrition guidelines. However, the AP had significantly higher protein content than recommended (up to 2.9 ± 0.5 g·kg^−1^·d^−1^; *p* < 0.001 for H male). The aim of this study was to quantify the environmental impact (EnvI) of the AP and to evaluate the influence of meal type, training load, sex and registered dietitian (RD). The nutritional contents of 216 APs created by 12 sport RDs were evaluated using Computrition Software (Hospitality Suite, v. 18.1, Chatsworth, CA, USA). The EnvI of the AP was analyzed by life cycle assessment (LCA) expressed by the total amount of food on the AP, kg, and kcal, according to the Swiss Agricultural Life Cycle Assessment (SALCA) methodology. Higher EnvI is directly associated with higher training load when the total amount of food on the plate is considered for E (5.7 ± 2.9 kg CO_2_ eq/day); M (6.4 ± 1.5 kg CO_2_ eq/day); and H (8.0 ± 2.1 kg CO_2_ eq/day). Global warming potential, exergy and eutrophication are driven by animal protein and mainly beef, while ecotoxicity is influenced by vegetable content on the AP. The EnvI is influenced by the amount of food, training load and sex. This study is the first to report the degree of EnvI in sports nutrition. These results not only raise the need for sustainability education in sports nutrition in general, but also the urgency to modify the AP nutrition education tool to ensure sports nutrition recommendations are met, while not compromising the environment.

## 1. Introduction

Everything we produce and consume has an impact on the environment. Due to its relevance, the environmental impact (EnvI) of food production has not only been a topic of interest in the scientific community, but also a cause for social mobilization. It has been reported that food production and processing have an impact on climate change, generating around 26% of total greenhouse gas emissions (GhGe), using 61% of fresh water and 38% of the global ice-free land surface [1,2]. Many factors influence the degree of EnvI along the food supply chain, including pre-production, such as agricultural inputs, agricultural production and food processing, and post-production, such as distribution, retail and waste [2,3,4]. Thus, each food product will have a different EnvI. For example, the production of animal proteins, such as red meat and dairy products, has higher GhGe, water and land use than plant-based proteins [5,6]. As a consequence, daily food choices have a direct impact on the environment [1,7]. Furthermore, food choices also have an impact on human health [8,9,10]. Red meat and processed meat have been associated with a higher risk of chronic disease and cancer [11], and a high consumption of processed food, rich in fat and sugar, is associated with a higher prevalence of obesity and diabetes in developed and developing countries [9,12]. Finally, climate change in itself (e.g., wild fires and air pollution) as well as other environmental impacts (e.g., pollutants) can also have an impact on human health [13,14]. 

Life cycle assessment (LCA) is one of the most frequently used methodologies to evaluate the EnvI of foods and diets across the food chain [15]. Life cycle assessment is a suitable methodology to identify priority areas, also called environmental hotspots. Hotspots can be found in the area of food choices, entire food groups, or in food processing. In the past, LCA studies have calculated the EnvI of single foods, highlighting the impact of meat, and especially red meat, on the environment [5]. Recently, LCA studies have focused on modeled diets or a typical dietary pattern [16,17,18]. Scarborough et al. 2014 evaluated different eating patterns and found a difference between high meat eaters and vegans (7.19 kg CO_2_ eq/d vs. 2.89 kg CO_2_ eq/d, respectively) [19]. Similar results were found in other studies [20,21,22,23]. Furthermore, most studies focused on the evaluation of only one single environmental issue (e.g., GhGe), with few studies using a more comprehensive approach that considers different EnvI categories (e.g., ecotoxicity, biodiversity, water and land use) due to the complexity of LCA methodology [15,24]. Whereas GhGe, land, and water use are more frequently captured in LCA, the topic of biodiversity is still difficult to measure. However, the alarming loss of plant and animal species [25] and its relationship to food production indicates that EnvI of human diets should also include biodiversity. Finally, it is also possible to evaluate EnvI through the planetary boundary framework [26], which attempts to examine human activity in reference to the boundaries of earth’s resilience. The framework considers the environmental limits within a safe operating space, while capturing multiple variables in one framework (e.g., climate change, biodiversity, land use, etc.). The framework highlights the importance of linking all human activities to each of the different planetary boundaries when assessing their impact, which also includes food production and consumption. Thus, similar to LCA, this framework can help to set planetary priorities related to dietary guidelines such as meat and dairy products. Taking a more comprehensive approach when studying the EnvI is recommended.

The trilemma of health, environment and diet has to be understood from a perspective that there is a sweet spot between meeting dietary recommendations for health (and performance), while not compromising life on Earth. The EAT-Lancet commission recently published a paper calling for the “Great Food Transformation” from sustainable production to healthful consumption, highlighting the importance to address this issue from both environmental and nutritional sciences perspectives together. “Win-Win-Win” solutions must be prioritized that promote co-benefits when healthy eating comes from sustainable production [7]. Most of the current literature has been focused on the general population, whereas the diets of athletes and active people have not yet been addressed. Athletes and active individuals are advised to consume more food, including protein, according to greater needs from physical activity and sports training [27]. While national dietary guidelines from various countries and world health organizations also recommend increasing physical activity for health and longevity, protein recommendations generally remain at conservative levels, except for older adults and in weight loss [28,29,30]. Considering the latter, it may be the diets of the “Healthy & Wealthy”, as indicated by Garnett (2016), that continue to omit considerations of EnvI, while over-emphasizing muscle mass, physique, and weight [31]. Thus, there is an urgency to adapt nutrition recommendations for active and athletic individuals and integrate the links among environment, health, and performance to promote “Win-Win-Win” solutions in these populations [32,33]. 

**Figure 1:**
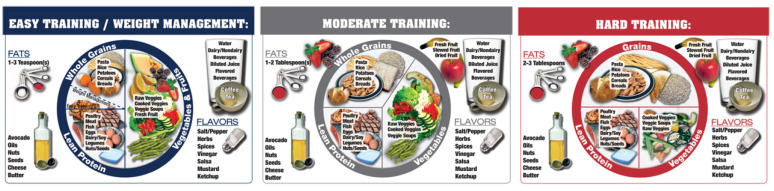
The Athlete’s Plate Nutrition Education Tool.

The Athlete’s Plate (AP) is a nutrition education tool specifically designed for athletes and active people. The aim of the AP is to help sports dietitians working with athletes or athletes themselves adjust food intake according to changes in training volume and intensity (defined in the training methodology literature as training load [34]). The AP is different from a nutrition education tool for the general population, as it adjusts the composition of the major food groups on the plate to training load without being too descriptive (see Figure 1). The AP supports the concept of nutrition periodization based on changes in training load throughout the annual training and competition plan. Following the concept of training load and nutrition periodization, three plates were designed: easy (E), moderate (M) and hard (H) training, and validated to ensure they meet international sports nutrition guidelines (see Reguant-Closa et al. 2019 for the details on the AP validation [35]). 

Sports nutrition recommendations for athletes are higher for energy and most macro- and micro- nutrients compared with the general population to ensure optimal health and adaptation to training so that performance capacity can be improved [27]. Specific guidelines exist to raise energy, carbohydrate, and fat intakes based on variable training loads. For protein, it is recommended that athletes consume between 1.2 and 2 g·kg^−1^·day^−1^, or 0.3 g·kg^−1^·day^−1^, which is 150–250% higher than the recommendation for the average person [27,36,37]. In some cases, higher amounts of protein have also been recommended [38] and identified in athletes’ diets [39]. Moreover, sports nutrition guidelines recommend high-quality protein sources that contain essential amino acids, especially leucine, to enhance muscle protein synthesis and promote repair of muscle tissues. For this reason, sports nutrition practice has been prioritizing animal and, specifically dairy protein, especially post-exercise [40]. Very little research exists on single or combined plant protein sources and their effect on muscle protein synthesis [41,42]. Hence, it is not surprising that a higher than recommended amount of protein, and especially animal protein, was also found when the AP was validated [35]. Knowing that animal proteins have a higher EnvI than plant proteins [1,6], it appears a prudent next step to investigate EnvI, alongside the health and performance effects, of athletes’ diets. Finally, it is well known that westernized countries consume more protein and, specifically more meat, than what is recommended [43]. Because protein recommendations for athletes are nearly two times higher than those for non-athletes, there is great concern that active, westernized populations consume protein, and specifically meat, in quantities beyond the need for optimal health, muscular development, and performance, while negatively impacting the environment. 

Thus, exploring environmental priority areas, including animal protein consumption, but also others, is the first step to evaluating EnvI of athletes’ diets, and specifically the AP model. Introducing changes to the AP, based on this study, will offer an evidence-based justification for making this educational tool specifically, and sports nutrition, more environmentally sustainable. This is the first study in sports nutrition that integrates LCA. Environmental research in sports nutrition is urgently needed, as recently highlighted by Meyer et al. 2020 [44]. 

Therefore, the purpose of this study was to quantify the EnvI of the AP and evaluate the influence of meal type, training load, sex and RD, to provide general recommendations to decrease the EnvI of the AP model and to make it more environmentally sustainable. This study is the first to explore EnvI in sports nutrition. We hypothesized that the AP’s EnvI will increase with training load and be higher than the EnvI of diets reported in the literature for the general population. We also hypothesized that the EnvI will differ among meals, sex, and RDs.

## 2. Materials and Methods 

This study assessed the EnvI of the AP created during the validation of the AP (see more detail described elsewhere [35]). Briefly, for the validation of the AP, 216 plates were analyzed. The plates were created by sport RDs familiar with the AP, addressing the following: differences in sex (females (F) and males (M)), meals (breakfast (B), lunch (L), and dinner (D)) and training load (easy (E), moderate (M), and hard (H)). Each RD reported to the dining hall at the Colorado Springs Olympic and Paralympic Training Center (CSOPTC) at separate times, without previously knowing the menu of the day. While adjusting their plate to the hypothetical scenario assigned, RDs were not confined to a plate or a dish but they could use all the dishes available, add more food to the same plate, use a plate or side bowl, or add a dressing, shake or dessert on the side. In total, each RD created 18 plates following the AP model for different training loads, meals, and sex. These plate data, made by RDs, were subsequently used to evaluate the EnvI of the AP using LCA. The methodological details regarding LCA are described below.

### 2.1. Life Cycle Assessment of the Athlete’s Plate

**Figure 2:**
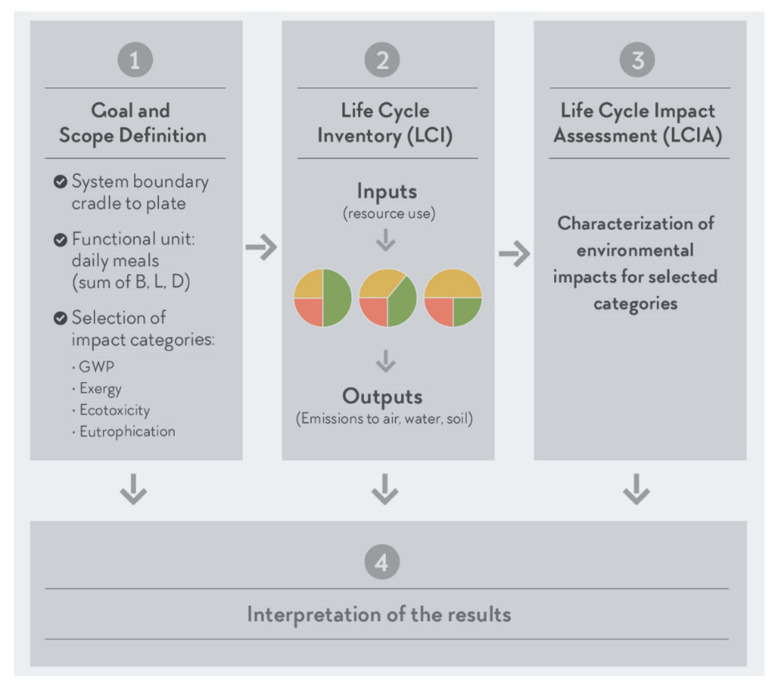
Diagram of the Athlete’s Plate (AP) life cycle assessment phases, with Phase 1 defining the goal (to evaluate the environmental impact (EnvI) of the AP) and scope of the study. The scope includes the system boundary which was defined from cradle to AP. Phase 1 also includes the choice of functional unit (FU), which expresses the data according to the goal of the study. This study used general FUs (per plate; per kg of food on the plate; per 1000 kcal) for comparison purposes. Phase 1 also includes the selection of EnvI categories quantified, which included global warming potential (GWP), exergy, ecotoxicity, and eutrophication. During Phase 2, which focuses on the life cycle inventory (LCI), all the inventories for the different foods were created depending on their inputs and outputs. Phase 3 which is the life cycle impact assessment (LCIA) in which the different inventories were analyzed for the EnvI categories selected. Phase 4 (Results) includes the interpretation of the results.

Life cycle assessment is a standardized methodology regulated by ISO 14040:2006, which includes four different phases to systematically evaluate the EnvI of a product or a service system through all stages of its life cycle [24,45]. To evaluate the EnvI of the plates created during the validation of the AP [35], an LCA was conducted. The food contents of each plate and detailed recipes were obtained from the AP validation [35]. For this LCA study, the Swiss Agricultural Life Cycle Assessment (SALCA) method was used [46]. Figure 2 represents a schema of the different LCA phases according to ISO 14040:2006. For a detailed update on LCA and food, see Nemecek et al. 2016 [15]. In the next sections, each of these phases are briefly described in more detail.

#### 2.1.1. Goal and Scope Definition

Phase 1 of LCA defines the goal of the study (what we want to analyze), the system boundary (inputs and outputs quantified within a selected boundary such as from cradle to plate), the functional unit (FU; how the EnvI data are expressed according to the goal of the study), and the EnvI categories studied. The goal of the current study was to analyze the EnvI of the AP created from the data of a previously published study [35]. 

**Figure 3:**
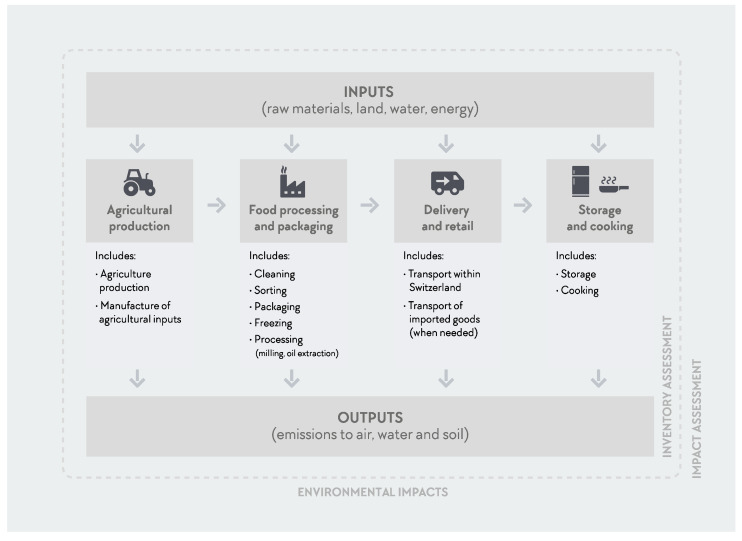
System boundaries of the Athlete’s Plate life cycle assessment used for the current study, ranging from production to consumer use. It includes all steps from agricultural production, processing, and packaging to transport, storage, and cooking.

The system boundaries included the agricultural production and all the downstream processes (post-farm processes) up to food preparation and the final AP using the ingredients available at the CSOPTC dining hall kitchen. The following phases were considered: agricultural production, including the manufacture of production means (like fertilizers, pesticides, fuels, etc.), processing and packaging, transport (within the country and imported transport distances and means when applicable), storage and cooking (when applicable). Pre or post-consumer waste was not included in this study. When a fresh product did not include any processing, for example, fresh apples, that phase/step of the inventory was considered zero. See Figure 3 for more details on the system boundaries of this study.

Environmental impacts in LCA are represented relative to an FU. Choosing the FU is a critical decision in LCA, as it affects the outcome and interpretation of the results [20,47]. Generally, the EnvI of foods is expressed per unit of product (grams) or calories (kcal). In LCA studies, with focus on nutrition and the human diet, the choice of FU depends on the goal of the study and, if selected relative to the food or nutrient function of interest, provides a more relevant representation of EnvI [48], although this is not always clear [49]. Only recently have FUs been specifically adapted to the functions of foods and nutrients, but most studies also provided EnvI using standardized units, so the study results may be compared [48,50,51]. In the AP model, each plate fulfills a pre-determined energy and nutrient need that maintains health and optimizes performance based on international sports nutrition standards, some of which are expressed relative to body mass (BM) at the 3 training loads (E, M, H). For this reason and for comparison purposes, the EnvI of the AP was expressed (1) per plate, (2) per kg of food on the plate, and (3) per 1000 kcal. 

Four environmental categories were selected to analyze the EnvI of the AP. Impact categories measure the EnvI of a product summing various substance emissions into one single measure to quantify their effect on the global or local environment. Most studies on LCA only focused on one EnvI category (e.g., GWP), but it is important to add multiple indicators to obtain a boarder representation of the impact. In this study, four EnvI categories were selected (see Table 1).

#### 2.1.2. Life Cycle Inventory (LCI)

Life cycle inventory is Phase 2 of the LCA analysis, where the inventories for each food/ingredient are analyzed according to the system boundaries defined in Phase 1. For each item, an inventory is created that includes all environmental inputs and outputs. This process, while complicated and extensive, is the core of LCA, as it determines the validity of the data. 

The quantification of the EnvI of food ingredients is very challenging due to the multitude of ingredients, origins, production and processing, and transport. The AP analyzed in this study included a wide variety of foods and ingredients. Some of the plates were composed with more than 100 ingredients. Due to the complexity of analyzing all the ingredients and to help simplify the analysis of the AP by LCA, assumptions had to be made and some ingredients were dismissed, aggregated or proxies were used. The dismissed products were those that represented a small portion of the plates (such as food additives, spices, some ultra-processed foods). Furthermore, where no data were available for some of the items analyzed, proxies were used. The different ingredients were aggregated into the following groups and subgroups: (1) dairy (milk, yogurt, soft cheese, hard cheese); (2) meat (beef, poultry, pork, processed meats); (3) eggs; (4) fish; (5) vegetables; (6) fruits; (7) grains (grains, breakfast cereals and bread); (8) legumes (all legumes except soy); (9) seeds; (10) sprouts; (11) nuts; (12) sugar (honey, sugar); (13) beverages (includes fruit juices and sweet beverages); (14) dressings (olive oil and mayonnaise based dressing) and (15) others. The other food categories included all ingredients that were only present on 1–2 plates in small quantities and inventories were not available or it was not possible to classify them in one of the previously described groups. An average value of all 13 groups was considered for the “others” group (see Table A1 for a detailed description of group aggregations). 

To achieve the goals of this study, detailed and specific LCA data were required to analyze the high variety of foods and products, which was not available for the US situation. It was therefore decided to analyze the data from a Swiss perspective, where access to more detailed data were available. Thus, for this study, the Swiss Agricultural Life Cycle Assessment (SALCA) method was used [46]. In this study, both primary and secondary data were used. The executive chef and manager of the CSOPTC Food and Nutrition Services provided the primary kitchen data through interviews. It included detailed ingredient composition for processed food and recipes, packaging, storage means and times, cooking means and times, details of procurement, and origin of products. Secondary data necessary to complete the inventories for each stage were obtained using the Ecoinvent (v3.4 cut-off by classification) and SALCA databases, supplemented with data from the literature when needed. Inventories were adjusted (such as: electricity mixes, transport distances and agricultural production) and adapted to the system boundaries of the study when needed (see Table A2 for a description of the assumptions). 

#### 2.1.3. Life Cycle Impact Assessment (LCIA)

Life cycle impact assessment is Phase 3 of an LCA and includes the characterization of EnvI for the different inventories created in LCI based on the selected EnvI categories (see Phase 1, Table 1). Inputs and outputs collected in the LCI phase were transformed into impacts. The SALCA 1.10 method was used to evaluate the results of this study. Impacts were calculated using SimaPro version 8.5.2.0 (PRé Sustainability, LE Amersfoort, The Netherlands).

#### 2.1.4. Statistical Analysis

Means and standard deviations were calculated for the EnvI of plates for all 12 RDs with the three training loads expressed (1) per plate, (2) per kg of food, and (3) per 1000 kcal. Pearson product correlations were calculated among the four EnvI indicators across RDs, training loads, sex, and meals to indicate the relationships (*n* = 216). Correlation analyses were also carried out among specific foods and GWP, exergy, ecotoxicity and eutrophication. Standard deviations within training load were combined (the three squared SDs per training load and RDs were averaged over the training load in order to estimate the between RD variation) for each RD to compare the variability in the four EnvI indicators across the 12 RDs. 

Sources of variation among RDs were investigated using a factorial treatment plan and repeated measures ANOVA in three separate analyses. The first analysis was with the main effect of RDs (*n* = 12), training load (*n* = 3) and sex (*n* = 2) using a 12 × 3 × 2 factorial plan. The second analysis was with RDs, meal and sex in a 12 × 3 × 2 factorial plan. The third analysis combined training load, meal and sex in a 3 × 3 × 2 factorial plan to test for second-order interactions. The 3 separate analyses were carried out because of the insufficient number of degrees of freedom for completing a 12 × 3 × 3 × 2 factorial analysis. 

Outliers were considered as a minimum of 3 standard deviations away from the mean and were evaluated by RD, meal and training load. Standard variations and coefficient of variation by training load for each RD were used to investigate individual variability of each RD. 

## 3. Results

### 3.1. Descriptive Data

To quantify the EnvI of the food content of the plates, a first data analysis was performed between training load and the four EnvI categories. The EnvI of the AP varied with training load. The results are shown in daily means ± SD for all meals together (i.e., breakfast, lunch, and dinner) at the three training loads (E, M, H) and expressed by the four EnvI categories (Table 2). Values are expressed by three different FUs, using absolute (per plate) and relative (per kg and 1000 kcal) values. These values reflect daily intakes, including the three main meals of the day (B, L, D) without snacks. 

A correlation analysis was performed in order to analyze synergies and trade-offs among EnvI categories and for specific food groups. A positive correlation indicates a synergy, while a negative correlation represents a trade-off. Correlation coefficients among the computed environmental indicators ranged from *r* = 0.86 to *r* = −0.05, with the higher correlations found between GWP and exergy (see Table A3). Due to the lower correlation among the different EnvI categories, results in the next sections are expressed by all four categories. Correlation analysis among the total EnvI of the AP and each food group category was performed for each impact category. Relatively high correlation coefficients were found between the total impact per plate and the impact for beef (GWP (*r* = 0.64); exergy (*r* = 0.70)). The same was found for the food categories of milk (GWP (*r* = 0.50); exergy (*r* = 0.48)) and eggs (GWP (*r* = 0.43); exergy (*r* = 0.40)). For ecotoxicity, higher correlations were found for vegetables (*r* = 0.73) and legumes (*r* = 0.64). For eutrophication, higher correlations were found for legumes (*r* = 0.55) and fish (*r* = 0.57). 

### 3.2. The Influence of Different Foods on the Total Environmental Impact 

**Figure 4:**
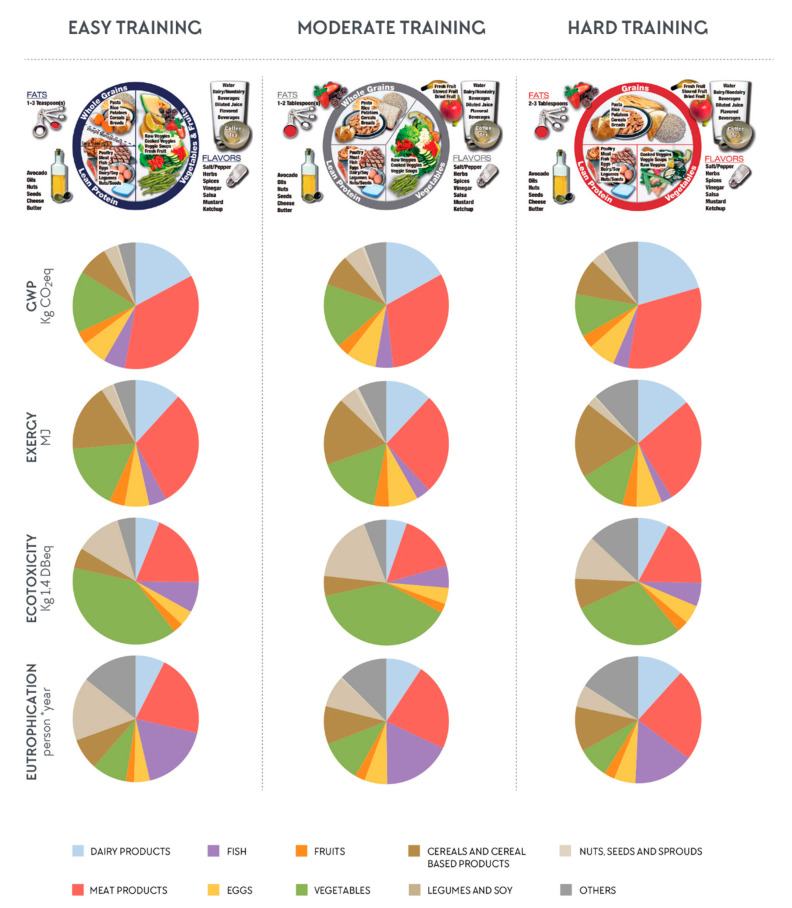
The contribution of each food group (aggregated) by training load and environmental impact category per plate. Figure 4 shows that meat and dairy contributed more to GWP and exergy, vegetables and legumes to ecotoxicity, and legumes and fish to eutrophication.

**Figure A1:**
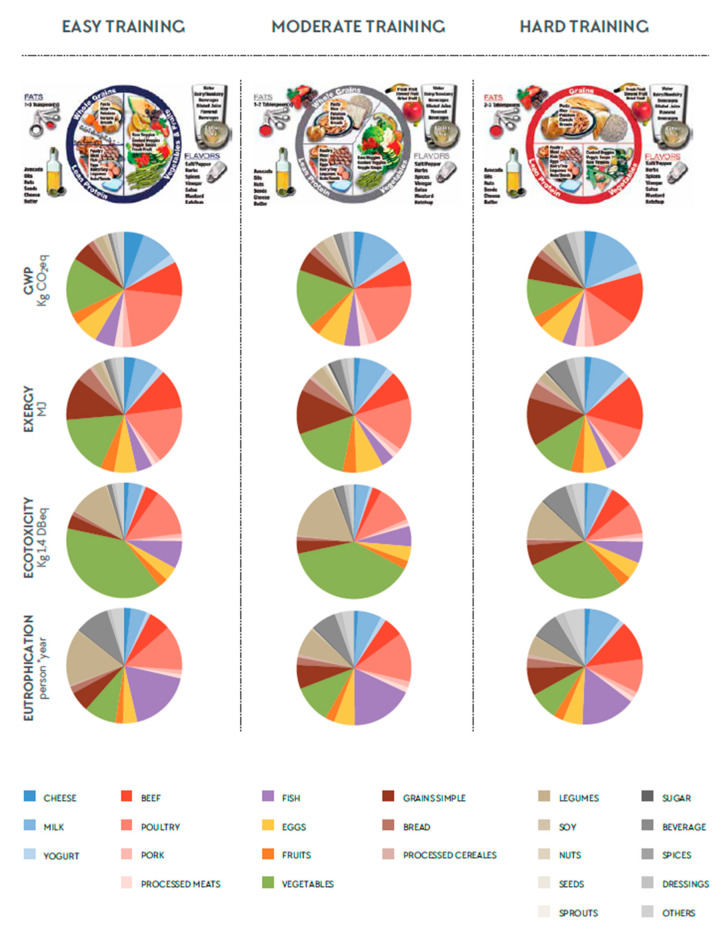
The contribution of each food group by training load and environmental impact category per plate.

In order to evaluate the contribution of each food group on the EnvI of AP as well as for each EnvI category studied, pie diagrams were developed for each training load (Figure 4). Pie diagrams are an ideal tool to provide an analysis of the contribution from each food group to the total EnvI of AP. Pie diagrams show that the total EnvI of the AP was affected more by meat and dairy than legumes and grains but the effect was specific to the EnvI category considered. For example, meat contributed more to GWP and exergy, vegetables and legumes to ecotoxicity, and legumes and fish to eutrophication (Figure 4). When evaluating each food group more closely, meats such as beef and chicken are the main contributors to GWP and exergy (Figure A1). When analyzing the plates individually, all plates with meat had a higher EnvI than those without meat (GWP = 2.5 and 1.6 kg CO_2_ eq respectively). If we consider different meat groups, the mean GWP values were higher when plates included beef (GWP = 3.6 kg CO_2_ eq) compared with pork (GWP = 2.9 kg CO_2_ eq) and poultry (GWP = 2.2 kg CO_2_ eq). Although some food groups had a higher EnvI than others, the total EnvI of the AP is always a result of a combination of the different foods groups. Moreover, the contribution of each food group to the total EnvI of the AP depends on the impact category evaluated. Figure 4 is a representation by EnvI category of the contribution of each food group by training load.

### 3.3. Sources of Variation among RDs

To evaluate sources of variation among RDs, meal type, training load and sex, a factorial treatment plan and repeated measures ANOVA was performed. ANOVA is used to verify if the mean EnvI differs among RDs, training load, meal and sex (main effects). Analysis I was based on a 12 × 3 × 2 factorial design to examine the main effects of RD (*n* = 12), training load (E, M, H) and sex (F, M). Analysis II (12 × 3 × 2) examined the main effects of RD, meal (B, L, D) and sex (M, F). And analysis III (3 × 3 × 2) tested training load, meal, and sex. All three factorial analyses were performed per plate. 

Analysis I showed a significant main effect on training load for GWP (*p* = 0.024), exergy (*p* = 0.018) but not for ecotoxicity (*p* > 0.05) and eutrophication (*p* > 0.05). Differences were also found between sex for GWP (*p* ≤ 0.05), exergy (*p* ≤ 0.05) and ecotoxicity (*p* ≤ 0.05), but not eutrophication, with males having a higher EnvI than females. There was a significant linear effect for training load for ecotoxicity (*p* ≤ 0.05) and exergy (*p* ≤ 0.05), but not for GWP or eutrophication (as training load increases, EnvI increases). There were no significant interactions for the four analyzed EnvI categories (*p* > 0.05). 

**Figure 5:**
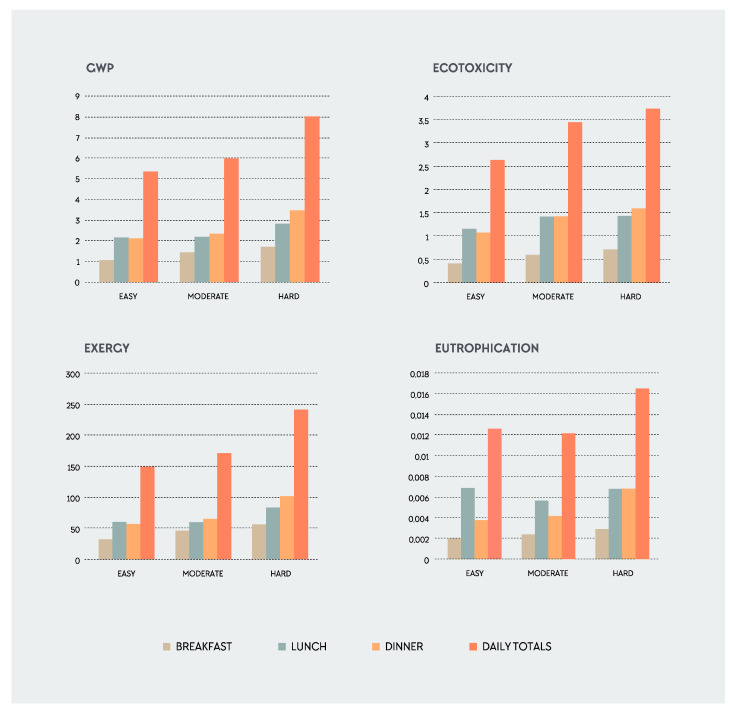
Meals and training load distribution of the four environmental impact categories. Values represented are per plate. Daily totals represent the sum of breakfast, lunch and dinner.

Analysis II showed a significant or almost significant quadratic effect of meal for GWP (*p* = 0.065), exergy (*p* = 0.166), ecotoxicity (*p* ≤ 0.05) and eutrophication (*p* = 0.047). This indicates that the EnvI values for breakfast are lower, while for lunch and dinner they are higher (Figure 5). In addition, statistically significant differences between sex were found for GWP (*p* ≤ 0.05), exergy (*p* ≤ 0.05) and eutrophication (*p* ≤ 0.05), but not for ecotoxicity. Males have a higher EnvI than females. For ecotoxicity, there is a significant effect for training load (*p* ≤ 0.05) and meal (*p* ≤ 0.001), with no effect for sex (*p* = 0.17). There was a significant linear effect of meal for GWP (*p* ≤ 0.05) and ecotoxicity (*p* ≤ 0.05), but not for eutrophication and exergy. 

**Figure 6:**
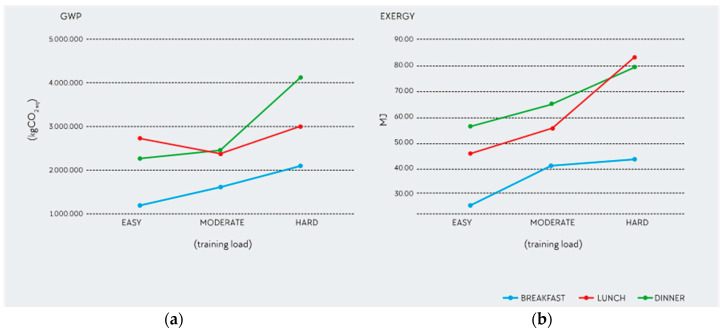
Two-way interaction for GWP and exergy: (**a**) **GWP**. Female two-way interaction (meal × training load) for GWP. (**b**) **Exergy**. Male two-way interaction (meal × training load) for exergy.

Analysis III was used to test for additional interactions without considering RDs. For both GWP (*p* ≤ 0.05) and exergy (*p* ≤ 0.05), there was a significant interaction among sex, meal, and training load, characterized by a non-parallel evolution of the interaction lines (Figure 6). 

The two-way interaction is significant for the two independent variables, training load and meal, for both GWP (*p* ≤ 0.05) and exergy (*p* ≤ 0.05), but neither for sex and meal nor for sex and training load. The former means that the impact of the variable meal on GWP and exergy depends on training load. This means that the interpretation of the main effect GWP and exergy is incomplete and/or mis- leading. 

### 3.4. Outliers and Consistency of the Data

**Figure 7:**
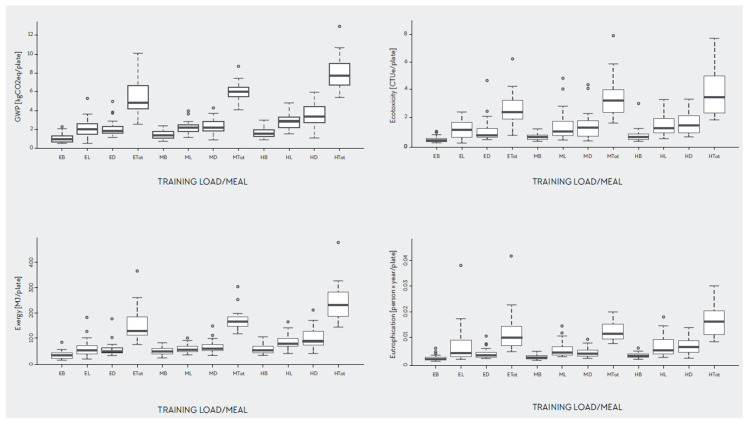
Boxplots by training load and meals for all four environmental categories. EB: Easy breakfast; EL: easy lunch; ED: easy dinner; Etot: easy daily totals; MB: moderate breakfast; ML: moderate lunch; MD: moderate dinner; Mtot: moderate daily totals; HB: hard breakfast; HL: hard lunch; HD: hard dinner; Htot: hard daily totals.

Figure 7 shows the distribution of the four EnvI categories studied divided by training load and meals. Some potential outliers were identified and checked for plausibility. In all cases, outliers occurred because of variations in meat on selected plates and/or the combination of foods high in EnvI. Furthermore, the outliers differed based on the EnvI categories. As a consequence, it was decided to keep outliers in the data (*n* = 216). 

Combined standard deviations were calculated to compare the variation within RDs when making the AP (Table 3). Registered dietitian six was the one with the lowest variation among the four EnvI categories followed by RDs four and seven. As shown above, in Section 3 of the results, RD twelve was more variable for all four EnvI categories than all other RDs. Registered dietitian 12 had an SD of 3.8 with a variance ratio of 14/2.6, or 5.4, compared to a typical SD of 1.6 (Table 3). 

## 4. Discussion

The purpose of this study was to quantify the EnvI of the AP and evaluate the influence of meal type, training load, sex and RD. This study is the first to explore EnvI in sports nutrition. The results of this study may lead to adjustments to the AP nutrition education tool to guide the development of the sustainable AP meant to be a visual tool for the diets of athletes and active individuals today and in future years. The findings show that the EnvI of the AP varies by training load, but this depends on the FU. The EnvI of the AP is mainly influenced by the total amount of food on the plate, the food group combinations, meal type (B, L, D), and RDs. 

### 4.1. Descriptive Data

As expected, Section One of the results shows that training load is the main factor influencing the EnvI of the AP for the four EnvI categories. When adjusted by weight of food (kg) or energy equivalent (kcal), the EnvI of the AP no longer rises with increasing training load. In fact, it is rather the opposite when using 1000 kcal as FU, showing similar or slightly lower EnvI at higher training loads. These differences are due to adjusting the quantity of food to a sole unit such as kg or kcal, giving rise to other aspects of foods, such as food composition on the plate. This emphasizes the necessity to express the values of EnvI relative to the most adequate FU. Several studies have highlighted the importance of using an FU that links nutrition and EnvI of diets [15,59,60,61]. While human diets fulfill many functions, one function is to supply a required number of calories and nutrients. In the case of the AP, the three different training loads provide different amounts of calories and macronutrient distributions and, as a consequence, fulfill a different training/competition function. Further research is needed to evaluate the EnvI of the athlete’s diet relative to nutrients essential for performance beyond energy (such as carbohydrate intake, protein quality, or iron). For example, the hard AP fulfills the function to replenish glycogen stores for intense training. Higher training loads require higher energy and carbohydrate intakes to adequately perform as well as replenish glycogen stores, as broadly described in the sports nutrition literature [27,62]. Athletes should obviously not consume less energy during hard training days for environmental reasons. They should focus on achieving greater energy and carbohydrate intakes, while meeting but not exceeding protein recommendations. A plausible solution to reducing the EnvI of the hard training day plate is to lower or eliminate the animal protein portion on the plate and focus on carbohydrates. Some studies highlighted how plant-based diets can be beneficial for athletes due to their higher carbohydrate content [63,64], as athletes often have carbohydrate intakes below recommendations [65,66]. Hence, making sports nutrition recommendations for hard training days or competition that promote optimal carbohydrate availability with less or no meat offer environmental protection and an evidence-based approach to performance enhancement. While the easy and moderate training day plates have smaller food quantities, they still incur a higher EnvI when containing meat. Thus, promoting plant-forward (e.g., flexitarian) [67] and plant-based meals across all training loads still helps athletes to achieve carbohydrate and protein intakes, while reducing the total EnvI of the AP. Athletes who balance energy expenditure from sport with sufficient calories, carbohydrate, and protein, as recommended, are at low to no risk for low protein intakes, even with a plant-based approach [68]. 

Gastrointestinal (GI) discomfort is a frequent issue in athletes, especially on hard/competitive training days [69,70]. Plant-based and forward strategies may lead to greater dietary fiber intakes, which could, at least temporarily, increase GI discomfort [69,70] in athletes. Sports dietitians should help athletes with the selection of more refined carbohydrate sources (e.g., white pasta) as recommended by the AP for hard training days. In addition, adopting a step-wise approach with unfamiliar plant-based foods (e.g., beans, grains, tofu, nuts, seeds), preparation (e.g., cooking, sprouting, fermenting), and timing of ingestion in training first before using them in competition is common practice in sports nutrition. Finally, food and eating are embedded in culture and tradition and eating before competing might also contribute to the athlete’s psychological preparation. The future of sports nutrition should therefore continue to focus on the individual, while gradually integrating environmental education of food choices when best suited.

The results of this study (reported as the sum of breakfast, lunch and dinner) show an average GWP for easy (5.3 ± 1.9 kg CO_2_ eq), moderate (6.0 ± 1.1 kg CO_2_ eq) and hard (8.0 ± 1.9 kg CO_2_ eq) days. To our knowledge, there are no previous studies that have quantified the EnvI of athletes’ diets or plates made according to changes in training load. In comparison with the general population, our results show a higher EnvI especially, but not only, for hard training days. Several studies show GhG emissions ranging from as low as 2.9 kg CO_2_ eq·d^−1^·person^−1^ (vegan) and up to 7.9 kg CO_2_ eq·d^−1^·person^−1^ (high meat eaters) using standard diets and food diaries [19,71,72,73]. Considering underreporting, even higher emissions are expected [71]. Methodological inconsistencies, such as the type of data sources, system boundaries, LCA methods as well as different dietary assessments used and populations studied, are common reasons for variability. The majority of studies have been conducted in non-exercising populations. There are currently no studies in athletes. Thus, the higher EnvI found in this study compared with other studies was expected, as the AP is targeted to athletes, meeting the needs of high energy expenditure. In addition, the results of this study are reported as the sum of breakfast, lunch and dinner, excluding snacks. Considering that snacks provide around 23% of the total daily energy intake in athletes [74,75], if anything, this study underestimates the EnvI of the AP. Different studies, addressing both the health and EnvI of diets in developed and developing countries, suggested a decrease in overall caloric consumption or at least energy density [12] for weight control and for the overconsumption of calories in westernized countries. Masset et al. 2014 studied French diets relative to calories and energy density and recommended both strategies to reduce the EnvI of diets [76]. These recommendations might be good for the general population. Athletes and active individuals need to consume more energy and nutrient-rich foods to match energy expenditure to daily training load. As a consequence, recommending foods with lower EnvI for individuals and groups who need to eat more food with greater energy and nutrient density should be done cautiously to ensure that daily needs are met and EnvI is not too high. 

A correlation analysis of the computed environmental indicators showed that the different indicators were not highly correlated, with the exception of GWP and exergy. Previous studies have shown that the strong correlation between GWP and exergy is related to similar key driving variables such as the use of fossil fuels [77,78]. Since not all EnvI indicators correlate but rather complement each other, all four should be included for a comprehensive picture of the EnvI of the AP, as has been recommended by others in LCA research [77].

### 4.2. The Influence of Different Foods on the Total Environmental Impact

Section Two of the results evaluated the contribution of each food group on the total EnvI of the AP. Whereas meat and dairy products show greater EnvI than legumes and grains, results vary by EnvI category and the types and quantities of food on the AP. Some food groups account for a greater EnvI of the AP than others, and thus, become more pressing environmental priorities (see Figure A1). Similar to other studies [8,19,23,79], the current results show that meat is the food group that accounts for the highest EnvI in all categories studied across all plates, except for ecotoxicity, where vegetables have a higher impact. All plates with meat had a higher EnvI impact than those without meat. The plates with beef (GWP = 3.6 kg CO_2_ eq) had a higher GWP than the plates with poultry (GWP = 2.2 kg CO_2_ eq) and double the GWP than the plates without meat (GWP = 1.6 kg CO_2_ eq). Poultry, without skin, is one of the most consumed meats among athletes, as it is lower in fat than red meat. It is known that red meat has a higher EnvI than poultry or pork, but because poultry is so frequently used by athletes, it plays a relevant role in the EnvI of the AP (Figure A1). Following meat, the dairy group also contributed to the EnvI of the AP. Dairy products are also frequently recommended to and used by athletes, especially for a meal addition or in recovery nutrition [80,81]. Most studies evaluating post-exercise protein synthesis used milk, whey, and casein as sources of high-quality protein. In the current study, dairy products showed high EnvI for GWP, exergy and eutrophication. For the AP, dairy products account for nearly 25% of the plate’s EnvI contribution to GWP. 

Sports nutrition recommendations include higher protein needs for athletes compared with the general population. In fact, protein recommendations are more than double for athletes. Moreover, in some specific cases, such as during energy restriction, even higher protein intakes are advised in the athletic population to avoid losses in lean mass [38,82], promote thermogenesis, and satiety [83,84]. Unfortunately, recommendations for athletes do not differentiate protein sources and generally focus on animal protein [40,85]. The AP validation study found that both moderate and hard plates exceeded protein recommendations for athletes. When protein sources were assessed, it was found that 70% of all protein originated from animal sources [35]. While several authors [32,33] have voiced concerns about such recommendations in the face of sustainability, no study has attempted to quantify the EnvI of athletes’ diets. Thus, this study is the first to report the degree of EnvI of athletes’ diets in general. We show higher GWP compared with the general population [40,80,82], pointing mostly to the contribution of animal protein to the AP’s EnvI. While high protein diets might seem an athletic issue, such diets are also recommended to the elderly to prevent sarcopenia [28,30,86] and are gaining popularity not only in weight loss but also to fulfill fitness and general wellness goals. Thus, the EnvI of these recommendations should be considered and integrated into future dietary guidelines for everyone. As described by Garnett (2016) [31] we need to find an equilibrium between nutrition recommendations, health and EnvI considerations globally and not only focus on the diets of the “healthy & wealthy”. 

While the quantity of animal protein has to be considered when discussing the impact on human health and the environment, with meat being a major contributor to environmental degradation and deterioration of health, the quality of the protein also matters. Protein quality impacts musculoskeletal development and athletic performance [37,87]. The aim of this study was to explore the EnvI of the AP made by sports RDs and the variability among training load, meal and sex and not to simulate the AP model to evaluate lower protein on the plate or substitutions for meat. This study also did not evaluate how substitutions for meat (e.g., with beans), while beneficial for the environment, would impact protein quality or performance. This is an area of needed future research. As previously mentioned, our concern relates more to the protein quantity of the AP and how educational efforts, in this case using the AP, integrate environmental causes. 

Regarding the quality of protein, different LCA studies have used protein and essential amino acids as FUs in the general population [51,61]. Berardy et al. 2019 studied LCA data relative to the digestible indispensable amino acid score (DIAAS) of single protein-rich animal and plant-based food sources [51]. They showed that nuts, beans, insects, fish or protein powders (from pea, soy or whey) provided the highest efficiency with the least EnvI, while beef, cheese, and some processed refined grains (such as rice or egg pasta) were the least efficient and had the highest EnvI. However, the list of products and serving sizes analyzed in the current study was not extensive enough (due to the lack of availability of some data) and we did not analyze the combination of different protein sources (animal with plant-based combinations) or culinary techniques that could complement or increase amino acid availability of plant-based sources [88,89,90]. In athletes, only limited research has integrated DIAAS of plant-based diets. Ciuris et al. 2019 [91] showed lower DIAAS and muscle mass and strength in endurance athletes on plant-based diets compared to athletes on meat-centric diets. Athletes are high users of protein supplements [92,93,94]. Protein powders have been shown to provide an efficient protein source with a good amino acid profile [51]. However, recent studies pointed toward the importance of whole intact proteins from milk or eggs, rather than their isolates (e.g., whey or albumin), for muscle protein synthesis [64,95]. Thus, it is unclear whether protein powders are the solution for both skeletal muscle and the environment. Finally, recent health concerns have also added reservations regarding protein supplementation in athletes [96]. Kårlund et al. 2019 studied the possible damaging effects of these products specifically, and high protein intakes in general, on the gut microbiome, suggesting possible negative consequences for both health and performance [96,97,98]. While still insufficient, studies are appearing on plant-based protein powders and combinations of plant-based protein foods and their effects on muscle recovery [41,42]. Furthermore, plant-forward strategies [67] and the addition of insects [99] might also be a possible strategy for active and athletic omnivores to integrate more environmentally friendly approaches [100] that also promote performance and health. 

In addition to meat and dairy, processed foods have a higher EnvI than unprocessed, fresh foods when expressed relative to GWP and exergy [101] and generally lower nutritional content [102]. The food groups with the highest EnvI in the current study were meat and dairy products. However, processed foods such as frozen and canned vegetables, fruits, and legumes, also contributed to the higher values for GWP and exergy. When fresh vegetables were compared with frozen or canned vegetables, GWP was 0.9 vs. 3.3 kg CO_2_ eq, respectively. Even though we did not evaluate the EnvI of each step of the food chain, the analysis of processed foods vs. unprocessed food inventories revealed significant differences. Vegetables were the main contributor to the EnvI for ecotoxicity. Ecotoxicity relates to intensive pesticide use in conventional agriculture. Taken together, conventionally grown and/or processed vegetables were both identified as an environmental priority in the current study. Integrating fresh and seasonal food decreases GWP and exergy, while organic vegetables would lead to lower ecotoxicity. 

Finally, total EnvI of the AP is determined by different food group combinations (Figure 4 and Figure A1). Based on Reguant-Closa et al. [35], the plate design might inadvertently lead to higher protein intakes because proteins from animal sources tend to be prioritized in sports, and milk is listed twice under protein and as a beverage. It also appears that the visual tool facilitates the identification of animal-based foods over plant-based alternatives, which may have contributed to the increased animal-based protein sources on the plates made by RDs [35] and the higher EnvI of the AP. A possible synergy to reduce the EnvI of the AP would be to decrease total meat and dairy content and increase plant-based options, including whole grains and legumes (not canned). Finally, APs with normal to high amounts of meat and/or dairy should not also include high amounts of plant-based proteins such as quinoa or legumes to avoid high EnvI from high-protein combinations. Such high protein combinations are most likely due to current food trends (e.g., quinoa) and menu design by All-You-Care-To-Eat dining. The sustainable AP will need to integrate these potential redundancy issues by an overall de-emphasis of animal-based proteins and promotion of plant-based and forward strategies.

### 4.3. Sources of Variation among RDs

Section Three of the results analyzed the main effects and interactions among RD, training load, meal, and sex using different factorial analyses. The results of this study confirm the hypothesis that the EnvI of the AP is dependent on training load. In addition, this study shows that EnvI varies according to meal, sex and RDs. When evaluating the effects of the different variables, interactions are important as they represent the combined effects of factors in the dependent measure. There was a significant main effect for training load and sex for all impact categories except for eutrophication. There was also a significant main effect for meal for all impact categories. Finally, the analysis shows that there was a significant interaction between RD and meal. This interaction indicates that the EnvI differs by RD when creating plates by meal type (B, L, D). Figure 6 shows the significant interaction between training load, meal, and sex for GWP and exergy for female and male, respectively, with greater EnvI for lunch and dinner and higher training loads. For meals, breakfast is generally smaller than the other meals, with less protein content compared with lunch and dinner [35], although this was not consistently the case in this study. Some breakfasts had higher EnvI (see Figure 7) than lunch or dinner. In athletes, breakfasts are not always the smallest meals. In fact, if breakfast is a pre-event meal, it might be larger than the other meals. Similar results were also found in other studies. Gillen et al. 2017 found a lower proportion of protein at breakfast (19%) compared with lunch (24%) and dinner (38%) [103]. In athletes, this could be explained by the periodization and distribution of food through the day relative to training sessions. Sports nutrition recommendations and the AP promote a diet lower in fiber and moderate in protein with higher amounts of refined carbohydrates, especially before training, with increasing training load to promote performance. This may be manifested by greater amounts of fiber and protein back-loaded at dinnertime compared with pre-training meals, such as breakfast and lunch. Athletes’ meal size, composition, and timing of ingestion vary greatly among sports and training phases, and thus, cannot be generalized as in the non-athletic population. Finally, in this study, breakfast was more plant-based or plant-forward compared to lunch and dinner, which included almost all meat portions consumed. This explains the lower EnvI of breakfast and reflects an opportunity for RDs to design and athletes to eat more plant-forward and plant-based lunches and dinners as a strategy to decrease the total EnvI of the AP.

### 4.4. Outliers and Consistency of the Data

Section Four of the results indicates the variability of RDs when creating the plates (Table 3). There is inherent variability in dietary data [104], which translates to the variability in EnvI data found in this study. In addition, data collection was performed in an “All-You-Care-To-Eat” dining hall with a high variability of foods from which to choose, which added another layer of variability. 

Sports nutrition recommendations are typically made in energy and nutrients and per kg of body mass but not specific to the types of foods, which is at the discretion of the athlete’s preference, the RD’s counseling practices and philosophy, and food services’ performance menu design. The AP is a visual tool but it is unclear which aspects are more important in guiding professionals and their athletes to adjust food intake to training load: the pictures on the plates, the colors, or the changing contribution of food groups. To our knowledge, there are currently no sports nutrition recommendations that integrate EnvI except for these articles [32,33,44], and the RDs in this study were trained in sports nutrition and not in the environmental aspects of food choices. The AP validation study showed that protein recommendations were exceeded for moderate and hard training loads [35]. Thus, RDs should not hesitate to tackle protein recommendations in practical settings, especially considering that athletes have sport science support teams who can monitor muscle mass and performance changes over time. Inter-professional research and collaboration could identify optimal protein intakes that still meet daily requirements but are not an environmental liability. In the future, training RDs about the EnvI of the food system, and specifically when teaching athletes which foods to choose and food service professionals in designing performance-based menus, will be an important strategy to integrate sustainability practices and ensure consistency among professionals.

### 4.5. Limitations

One limitation of this study is that we did not quantify organic vs. conventional farming systems. Whereas animal protein has a high impact on the plates for GWP, exergy and eutrophication, vegetables have a higher impact on ecotoxicity, as shown in Figure 4. This is mainly due to the use of pesticides in conventional compared to organic production; the latter results in lower ecotoxicity [56,105]. The fact that some of the products used at the kitchen of the CSOPTC are organic (rice, legumes, some fruits and vegetables, etc.) but were not accounted for in this analysis would have resulted in lower ecotoxicity from the vegetable category. Further LCA studies for meals and diets in the general and athletic population should include the impact of different production systems. Beans also had a higher impact in comparison with other studies [6], due to the fact that most beans used were canned. Thus, preference should be given to fresh, seasonal fruit and vegetables and dry beans. 

While this study tried to quantify the EnvI of real plates for athletes made by RDs following the AP model, developing the inventories for this LCA study was complicated and had some limitations. First, the list of ingredients considered for this study was very broad and different assumptions were made (Table A2). For the analysis, we needed a large number of inventories, covering a wide range of different food products and sufficiently detailed to represent the EnvI in the best possible way. Such a comprehensive dataset was not available for the US case, corresponding to our list of ingredients, so we decided to adapt the study to Switzerland because Swiss data, which fulfilled these criteria, were available. Efforts were made to create a consistent data set. Therefore, LCI data not developed for Switzerland were adapted to the Swiss case (e.g., transport, electricity mixes) and the system boundaries of this study, while the data analyzed regarding the composition of the AP were based on the AP made in the dining hall of the CSOPTC. This presents a limitation of the study, but institutional food service facilities share many similarities. Additionally, sports nutrition recommendations are similar around the world, with staple foods recommended worldwide for athletes (e.g., pasta, rice, chicken or yogurt among others). However, we should assume some possible impacts on the results of this study, had US LCI data been used. First, the electricity mix in the US is based more on fossil fuel, which would have increased GWP and exergy, especially for food transformation and storage processes (e.g., cooking and freezing) [106]. Second, longer transport distances are expected within the US, which also increases GWP and exergy [107]. Differences in other impact categories could be expected, but are unknown and this study did not analyze them. 

### 4.6. Recommendations

**Figure 8:**
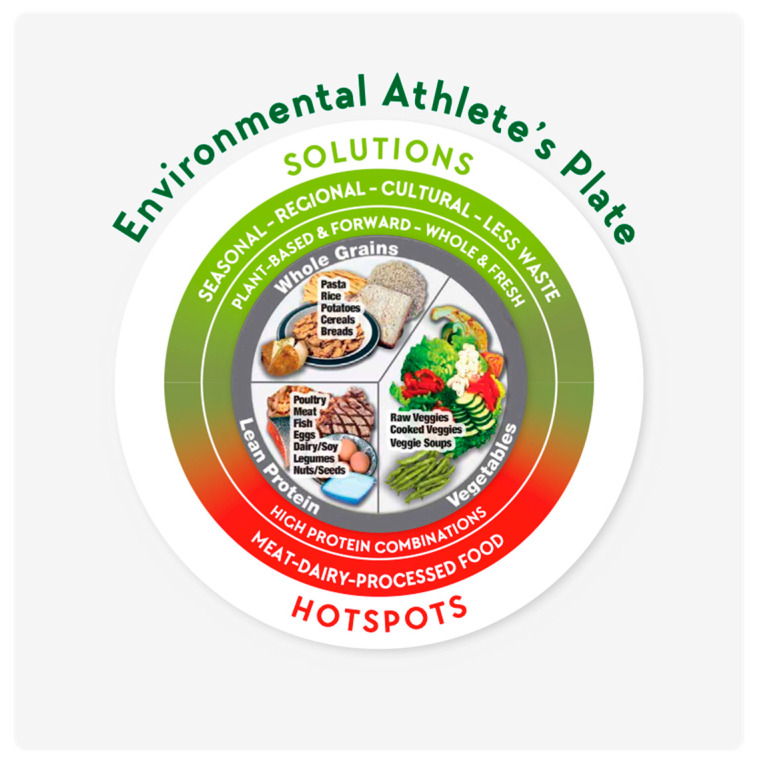
The Environmental Athlete’s Plate.

It was beyond the scope of this study to model an environmentally sustainable AP. Nevertheless, the findings allow for the identification of environmental priority areas (also called hotspots) that enable opportunities for education. To make changes and lower EnvI of the AP (and athletes’ diets), Figure 8 shows hotspots and possible solutions. To promote environmental sustainability in athletes we recommend athletes: (1) adjust energy, carbohydrate, and fat intake to the recommendations according to training loads, (2) reduce protein intake to the recommended level, (3) replace some animal protein with plant protein, (4) within the animal protein fraction, prioritize milk, eggs, poultry and pork over ruminant meat and cheese, (5) use fresh, seasonal, regional, and unprocessed foods and (6) limit frozen and canned products and reconsider protein powders that result in protein surplus, (7) obtain education in environmental issues of food choices when creating plates and (8) consider individual and cultural preferences.

Taken together, adjusting the AP, in combination with training in environmental nutrition and integration into dietetics practice and menu design (e.g., food combinations), is a necessary next step to address environmental sustainability in sports nutrition. A recent paper by the EAT-Lancet Commission 2019 urged for the “Great Food Transformation”, recommending a reference diet with an average of approximately 300 g of meat per person per week for human and environmental health [7]. Athletes may exceed this number on a daily basis. Thus, the current data should raise urgency and awareness in athletes, teams, and organizations that food for athletes impacts the environment, especially at higher training loads and if rich in meat and dairy products. Besides greater energy and nutrient intakes, athletes also travel frequently, use new and diverse equipment, and have a high consumption of packaged foods. While certain aspects of being an athlete cannot easily be modified, changing dietary habits not only for health and performance, but also the environment, presents synergies that are urgently needed.

## 5. Conclusions

To our knowledge, this is the first study to address EnvI in sport nutrition. The results of this study show that the EnvI of the AP is influenced by the amount of food on the plates but dependent on the FU. Moreover, the EnvI of the AP is influenced by the EnvI category analyzed and the food group combination, increasing when multiple high protein foods (especially meat and dairy) are included on the same plate. In addition, the analysis showed a significant main effect between training load and meal for almost all impact categories, with a higher EnvI for lunch and dinner and for the hard training plate. As the recommendations from this study point to a reduction in animal protein, with a subsequent increase in plant-forward and plant-based strategies, more research is needed on these substitutions in athletes. Reducing the amount of total protein, and especially animal protein, on the plates to the recommended intake, and including occasional plant-based meals, also in omnivores, will lower the EnvI of the AP. Recommendations for athletes and active individuals cannot be exempt from the urgency to address the sustainability of diets. This study showed high variability in the EnvI of the AP among RDs, which highlights the opportunity for education to decrease the EnvI of the AP, while maintaining an adequate diet for sport performance and health. Finally, this paper highlights the importance of trans- and interdisciplinary collaborations, not only among sport and health professionals, but also nutrition and environmental scientists, as this is needed in the twenty-first century.

## Figures and Tables

**Table 1:** Description of the environmental impact categories.

Environmental Impact Categories	Description
GWP (kg CO_2_ eq)	- GWP is the potential effect of GhGe on the climate. - Main GhG contributing to GWP of food systems are: carbon dioxide (CO_2_), methane (CH_4_) and nitrous oxide (N_2_O). For industrial processes some hydrocarbons can also contribute. - The use of fossil fuels, ruminant production, use of nitrogen fertilizer or organic matter decomposition, increase GhGe [52].
Exergy (MJ)	- Considered as the use of all renewable and non-renewable resources that are used when making a product. - Exergy covers the use of land, water, energy carriers (renewable and non-renewable), minerals, metals, as well as biomass extracted from natural systems (e.g., during deforestation) [53,54].
Ecotoxicity (kg 1,4DB eq)	- Represents the effect of a substance on the environment and on human health [55]. - The toxic effect of a substance depends on its environmental chemistry (exposure) and the effects of the substance on the organisms that come into contact with it. - Human toxicity is generally affected by the use of pesticides or heavy metals mainly included in the phosphorous fertilizers [56,57].
Eutrophication (aquatic and terrestrial) (person × year)	- Represents aquatic and terrestrial accumulation of nitrogen and phosphorus from application of excess fertilizer with subsequent agricultural runoff. Leads to algae growth and oxygen deficiency in marine environments [58].

GWP: global warming potential; GhGe: green house gas emissions; units for each environmental impact category in parenthesis are defined as: GWP (kilogram equivalents of carbon dioxide); exergy (megajoule); ecotoxicity (kilograms of 1,4 dichlorobenzene equivalents); eutrophication (person × year).

**Table 2:** Athlete’s Plate daily totals (sum of breakfast (B), lunch (L), and dinner (D)) by training load.

		Easy	Moderate	Hard
		Mean ± SD (*n* = 72)	Mean ± SD (*n* = 72)	Mean ± SD (*n* = 72)
**Total food weight (kg)**		2.6 ± 0.6	2.4 ± 0.7	2.7 ± 0.7
**GWP** (kg CO_2_ eq)	**Per Plate**	5.3 ± 1.9	6.0 ± 1.1	8.0 ± 1.9
	**Per kg**	2.6 ± 0.8	2.5 ± 0.4	2.5 ± 0.3
	**1000 kcal**	3.1 ± 1.0	2.6 ± 0.5	2.5 ± 0.5
**Exergy** (MJ)	**Per Plate**	149.0 ± 64.1	171.0 ± 40.1	241.1 ± 73.7
	**Per kg**	72.8 ± 26.2	71.9 ± 19.6	73.7 ± 13.6
	**1000 kcal**	84.7 ± 25.2	75.1 ± 17.4	76.1 ± 16.2
**Ecotoxicity** (kg 1,4DB eq)	**Per Plate**	2.6 ± 1.2	3.4 ± 1.6	3.7 ± 1.6
	**Per kg**	1.3 ± 0.5	1.4 ± 0.5	1.1 ± 0.4
	**1000 kcal**	1.6 ± 0.9	1.5 ± 0.7	1.2 ± 0.6
**Eutrophication** (person × year)	**Per Plate**	0.0125 ± 0.0083	0.0121 ± 0.0037	0.0165 ± 0.0061
	**Per kg**	0.0079 ± 0.0079	0.0056 ± 0.0023	0.0058 ± 0.0028
	**1000 kcal**	0.0077 ± 0.0061	0.0053 ± 0.0016	0.0053 ± 0.0018

Units for each environmental impact category in parenthesis are defined as: Global warming potential (GWP; kilogram equivalents of carbon dioxide); exergy (megajoule); ecotoxicity (kilograms of 1,4 dichlorobenzene equivalents); eutrophication (person × year). Data represent environmental impact categories for easy, moderate, and hard training loads.

**Table 3:** Standard deviation of the environmental impacts of the 12 dietitians (easy, moderate and hard plates) for daily totals (sum of breakfast, lunch and dinner).

DIETITIAN	GWP (kg CO_2_ eq)		EXERGY (MJ)		ECOTOXICITY (kg 1.4DB eq)		EUTROPHICATION (person × year)	
	Mean	SD	Mean	SD	Mean	SD	Mean	SD
1	6.1	2.2	180.6	64.9	2.8	1.0	0.0155	0.0033
2	6.0	1.9	155.0	27.2	4.1	1.5	0.0134	0.0059
3	6.3	1.4	162.3	29.3	2.8	0.7	0.0090	0.0019
4	6	1	169.2	19.2	3.2	1.1	0.0127	0.0023
5	6.3	1.7	169.3	42.2	3.4	1.7	0.0182	0.0137
6	4.9	0.6	140.4	11.7	1.9	0.5	0.0086	0.0008
7	5.8	1.0	157.5	25.8	2.3	0.4	0.0111	0.0044
8	7.3	1.1	194.1	31.5	3.4	0.6	0.0114	0.0023
9	6.0	1.5	183.8	70.0	3.1	1.4	0.0165	0.0055
10	7.3	1.8	208.8	45.6	4.1	1.5	0.0165	0.0057
11	7.2	1.4	214.3	41.6	5.4	1.7	0.0188	0.0054
12	8.0	3.8	309.1	114.7	2.7	1.0	0.0130	0.0059

Means and combined standard deviations (obtained by calculating the three squared SDs per training load and RD and averaged over the training load in order to estimate the between RD variation). Means and SDs were calculated to compare variability in the four environmental indicators across the 12 RDs. Units for each environmental impact category in parenthesis are defined as: GWP (kilogram equivalents of carbon dioxide); exergy (megajoule); ecotoxicity (kilograms of 1,4 dichlorobenzene equivalents); eutrophication (person × year).

## Appendix A

**Table A1:** Food group aggregations.

AGGREGATION OF FOODS	GROUPS INCLUDED	SUBGROUPS
**DAIRY**	Milk products	-
	Yogurt	-
	Cheese products	Soft cheese and hard cheese
**MEAT**	Beef	-
	Poultry	-
	Pork	-
	Processed meats	-
**FISH**	Fish	-
**EGGS**	Egg	-
**VEGETABLES**	Fresh vegetables	Specific inventories for vegetables where created when possible
	Canned vegetables	Specific inventories for vegetables where created when possible
	Frozen vegetables	-
**FRUITS**	Fresh fruits	Specific inventories for vegetables where created when possible
	Frozen fruits	Specific inventories for vegetables where created when possible
**GRAINS**	Grain simple	Includes pasta, rice, bulgur, oats, etc.
	Bread	-
	Processed cereals	Includes breakfast cereals with sugar and processing
**LEGUMES**	Legumes without soy canned	-
	Soy beans	-
	Tofu	-
**NUTS**	Nuts in general	-
	Peanuts	-
	Peanut butter	-
**SEEDS**	Seeds	-
**SPROUDS**	Sprouts	-
**SUGAR**	Honey	-
	Sugar	-
**BEVERAGES**	Bottled fruit juice	-
	Wine	-
	Sports drinks	-
**SPICES**	Spices	-
**DRESSINGS**	Olive oil vinaigrette dressing	-
	Mayonnaise based dressing	-
**OTHERS**	Others	-

**Table A2:** Athlete’s Plate study LCA Assumptions.

STAGE	ASSUMPTIONS
**Agriculture production**	- When a product is produced in Switzerland (ex. Apples) agricultural production in Switzerland was selected. Existing inventories from ecoinvent or SALCA were selected. - When a product is not produced in Switzerland (ex. avocado) main importing countries are considered.
**Processing**	Due to the difficulty of accounting of all the individualities of processing steps due to the wide range of ingredients considered for this study, some assumption were considered on this life-cycle stage: - Existing ecoinvent inventories were used for processing (including: cleaning, sorting, milling, cheese making, sterilization, freezing process, etc.) - In highly perishable vegetables and fruits (example: berries) adapted inventories were used due to the difference of manufacture. - In some cases, storage time of fresh product in the processing plant and short transport distances between plants (20 km) was included on the processing inventories of ecoinvent. Those were also included in this study. - When the inventory was not specific to Switzerland, electricity mixes were adapted to Switzerland.
**Packaging**	- Packaging was in some cases already included in the processing inventories from ecoinvent. - When it was not included, it was added. - When specific packaging data were available (type, weight, etc.) from the primary data collected during the study in the kitchen, packaging was adapted to this specific case.
**Transport**	- Transport distances inside Switzerland are assumed to be an average of 100 km. 50 km by train and 50 km done by lorry. When frozen or cooled products were considered, transport means included frozen or cold conditions. - Import transport distances and means were added when there was not production in Switzerland based on main importing countries to Switzerland. Inventories from ecoinvent were considered.
**Storage**	- Added storage time was considered for fresh products at the kitchen and was added on the inventories. Time of storage was divided into freezer and fridge depending on the product. Time of storage varies from 3 days up to 6 months depending on the product and was adapted individually for each inventory depending on the primary data collected at the kitchen. - Electricity mixes were adapted to Switzerland. - Dry storage was not considered.
**Cooking**	- Cooking inventories available at ecoinvent were used. - Cooking times and means (oven, stove, etc.), were adapted to each particular case following primary data collected at the kitchen and the recipes available. - Electricity mixes were adapted to Switzerland.

**Table A3:** Correlation of the four environmental indicators by daily totals (sum of B, L, D) and by plate.

	GWP (kg CO_2_ eq)	Exergy (MJ)	Ecotoxicity (kg 1.4DB eq)	Eutrophication (person × year)
GWP	1	0.86	0.69	0.26
Exergy	0.86	1	0.45	0.28
Ecotoxicity	0.69	0.45	1	0.44
Eutrophication	0.26	0.28	0.44	1
